# Writing in the Air: Contributions of Finger Movement to Cognitive Processing

**DOI:** 10.1371/journal.pone.0128419

**Published:** 2015-06-10

**Authors:** Yoshihiro Itaguchi, Chiharu Yamada, Kazuyoshi Fukuzawa

**Affiliations:** Psychology Section, Faculty of Letters, Arts and Sciences, Waseda University, Tokyo, Japan; University of Leicester, UNITED KINGDOM

## Abstract

The present study investigated the interactions between motor action and cognitive processing with particular reference to kanji-culture individuals. Kanji-culture individuals often move their finger as if they are writing when they are solving cognitive tasks, for example, when they try to recall the spelling of English words. This behavior is called *kusho*, meaning air-writing in Japanese. However, its functional role is still unknown. To reveal the role of kusho behavior in cognitive processing, we conducted a series of experiments, employing two different cognitive tasks, a construction task and a stroke count task. To distinguish the effects of the kinetic aspects of kusho behavior, we set three hand conditions in the tasks; participants were instructed to use either kusho, unrelated finger movements or do nothing during the response time. To isolate possible visual effects, two visual conditions in which participants saw their hand and the other in which they did not, were introduced. We used the number of correct responses and response time as measures of the task performance. The results showed that kusho behavior has different functional roles in the two types of cognitive tasks. In the construction task, the visual feedback from finger movement facilitated identifying a character, whereas the kinetic feedback or motor commands for the behavior did not help to solve the task. In the stroke count task, by contrast, the kinetic aspects of the finger movements influenced counting performance depending on the type of the finger movement. Regardless of the visual condition, kusho behavior improved task performance and unrelated finger movements degraded it. These results indicated that motor behavior contributes to cognitive processes. We discussed possible mechanisms of the modality dependent contribution. These findings might lead to better understanding of the complex interaction between action and cognition in daily life.

## Introduction

Kanji (a Japanese logographic symbol representing a lexical morpheme) culture individuals often show a particular behavior when they deal with their written languages; they move their index finger as if they were writing mysterious codes in space or on a table, especially when they count the number of strokes of a character or when they try to recall the shape of a kanji or the spelling of English words. Such behavior is called *kusho*, meaning air-writing in Japanese [1–3]. The number of children using kusho increases with age, and even individuals from non-kanji cultures show kusho behavior if they spend a number of years in contact with kanji [1]. These phenomena suggest that repeated writing in the learning phase of kanji leads to the use of such a strategy. Although it has been almost 30 years since the first report of kusho [1] was published, the functional role of the finger writing movement in cognitive process is still not clear.

**Fig 1:**
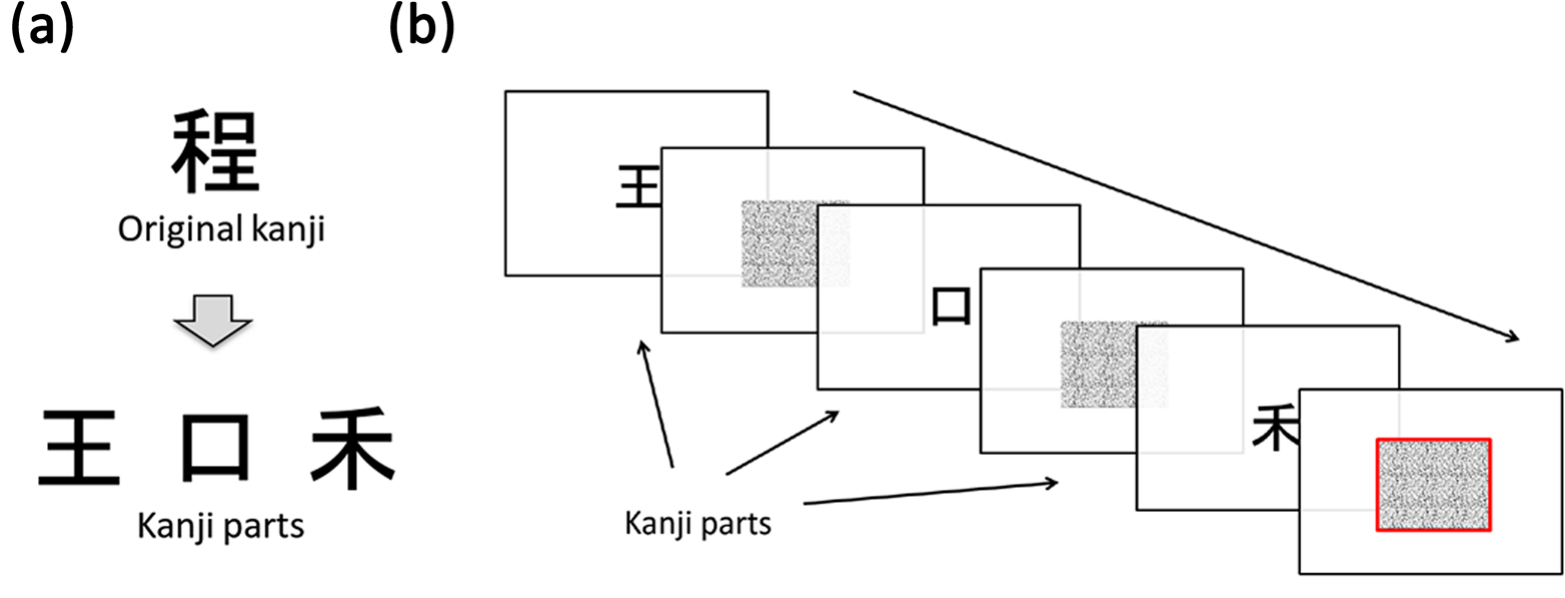
Experimental stimuli and sequence. (a) An example of an original kanji and kanji parts. Usually, kanji are made up of a number of commonly used parts. (b) Time sequence of the construction task in Experiment 1.

Kusho behavior is thought to facilitate visual processing in solving cognitive tasks. Sasaki and his colleagues examined the facilitation effect of finger writing movement on performance in a kanji construction task [1]. In their experiment, participants tried to put several components (graphemes) of a kanji character together in their mind (Fig 1A), which were presented either visually or verbally. In both visual and verbal presentations, the participants made more correct responses when they were allowed to use finger movements than when they were not allowed to. The study further demonstrated that finger writing on a surface such as a table improved performance of the task much more than writing in the air, suggesting that kusho is involved with visual processing in cognitive tasks. This effect was observed in both Japanese children and Chinese students, providing evidence of the generality of the proposed kusho function [1].

On the other hand, neuropsychological symptoms highlight the importance of the kinetic aspects of finger movement on letter and character recognition. Pure alexic patients often use finger tracing movement as an aid in reading characters which they are otherwise unable to read (kinesthetic facilitation) [4–9]. By contrast, agraphia patients cannot make use of kinetic movement to identify characters without seeing them [10–11]. Based on an examination of agraphia patients, Fukuzawa et al. [12] suggested that retrieval of the visual image of letters from memory occurs before the initiation of writing. Thus, it is likely that the kinetic aspects of finger writing may elicit motoric representations of letters and characters, resulting in improvement in reading and recognition [1,3].

The kinetic aspects of finger movements can also facilitate solving arithmetic tasks. A number of studies have shown that finger movements interacted with execution of arithmetic tasks [13–16]. Moreover, Michaux et al. [17] showed that unrelated finger movements interfered with addition and subtraction, but not with multiplication. The results could be interpreted by postulating that adding and subtracting require a procedural process, while multiplication does not (finger-based vs memory-based) [17]. Imaging studies also reported increased activity in motor-related areas during arithmetic tasks [18–19]. These findings imply that over-learned movements can influence cognitive processes, in agreement with the interaction effects of kusho behavior on cognitive tasks.

Nevertheless, we still know little about the role of kusho behavior in cognitive tasks. Although Sasaki [1] showed a facilitative effect of kusho behavior on correct responses in a kanji construction task, the study did not separate possible effects from visual and kinetic aspects of kusho behavior. Not only visual but also kinetic aspects of kusho behavior provide sensory feedback and motor commands (afferent and efferent information). This kinetic information could elicit motoric representations or engrams of a character [1,20–21]. To discuss the kinetic effects of finger movement, Matsuo et al. [22] investigated the neural activation of the brain using fMRI. They found that neural activity in the dorsal occipitoparietal area and the primary visual area decreased when participants moved their index finger to count the strokes of kanji. The study implies that finger movement imitating writing could function to decrease neural loads on cognitive processing.

To further investigate the role of kusho behavior in cognitive tasks, the present study experimentally isolated visual from kinetic aspects from each other in finger movement. First, we measured the occurrence rate of kusho behavior in cognitive tasks, using construction and stroke count tasks (Experiment 1). We then examined the visual and kinetic effects of finger movements on cognitive tasks (Experiment 2 and Experiment 3). Finally, we tested the possibility that kinetic aspects of finger movement influences performance in cognitive tasks by using different versions of the tasks (Experiment 4).

We employed two different cognitive tasks, a construction task and a stroke count task. The construction task resulted in greater activation in the bilateral parieto-occipital area, the premotor area and the supplementary motor area than in simple writing, suggesting that the task required greater neural resources for visuo-spatial processing [23]. In contrast, counting strokes of a character activated the premotor area [22], which may be involved in the sequential processing of making one-to-one correspondences [13,24]. We added a new hand condition which was not employed in the previous study [1]. In this condition participants had to solve the task with an unrelated finger movement. We assumed that this movement requires unrelated motor commands and provides unrelated sensory feedback to participants. If there is a direct relationship between the representation of a character and its kinetic information, unrelated signals do not affect performance, or rather disturb it. By adding this condition, the present study could test both facilitation and interference effects in order to discuss the nature of finger movement effects on cognitive processing.

We examined two hypotheses which assumed that possible kusho effects occur at either visual or kinetic levels. First, if the kinetic aspects of finger movements corresponding to a specific character selectively contribute to cognitive processes, kusho behavior would improve task performance while unrelated movements would degrade it, irrespective of visual conditions (kinetic facilitation hypothesis). Second, if kusho behavior has a role in the visualization of finger movements, the effects would be observed only when participants see the movements (visualization hypothesis). We tested these hypotheses by conducting a series of four experiments.

## Experiment 1

In Experiment 1, we confirmed whether or not young Japanese people today demonstrated spontaneous kusho behavior in cognitive tasks, using the construction task and the stroke count task.

### Methods

#### Participants

Fifteen right-handed students participated in Experiment 1 (19.7 ± 0.5 years old). They were all Japanese native speakers and had completed their elementary to high schooling in Japan without any problems in reading and writing Japanese characters.

#### Stimulus

The same sets of kanji stimuli were used in the construction and the stroke count tasks, but the ways of presenting them were different. In the construction task, three subparts of the kanji were presented successively, while in the stroke count task a whole kanji character was presented. A total of 66 kanji stimuli were selected from the 1,006 kanji which Japanese children learn in elementary school. The kanji selected could be broken down into three parts (Fig 1A) and contained no parts which had the same reading as the original kanji [1]. One or several parts of a kanji can also be independent kanji characters, which therefore have their own readings and meanings. For example, in Fig 1A, the original kanji is composed of three parts, two of which are other kanji characters and accordingly have other readings. The original kanji can be read as /tei/ or /hodo/, and the kanji parts are /ou/ and /kou/ or /kuchi/ respectively. To avoid the possible facilitation effect of the same reading between the original kanji and its parts, the present study controlled the phonological variable.

Three sets of kanji stimuli were created so as to have the same character properties on average: familiarity [25], complexity [26], grade level learned in school, and difficulty. The difficulty was defined as the percentages of correct responses and response times obtained from a preliminary experiment using another 8 different participants (22.3 ± 1.5 years old). The grand means of the familiarity, the complexity, grade level, percentages of correct responses and response times were 5.43 ± 0.68, 4.50 ± 0.64, 4.11 ± 1.30, 0.57 ± 0.23%, and 3.88 ± 2.02s respectively.

#### Construction task

Participants were asked to read aloud the original kanji character as quickly as possible after the subparts of the kanji were presented on a screen; they constructed a kanji character using the presented three subparts like a puzzle. The kanji parts, about 6×6 cm in size and positioned at the center of the screen, were successively presented 1s after each other. A mask image (random dots) was interpolated between each stimulus for 0.5s. After the last stimulus was presented, a mask image with a red borderline appeared and remained for 10s, during which time the participants were asked to answer into a microphone in front of them (Fig 1B). They were also instructed not to answer before the last stimulus disappeared even if they could, because measurement started from the ending of the last stimulus. When the participants tried to reconstruct a character, they could overlap, expand, or reduce in size the presented parts to form a kanji character in their mind.

#### Stroke count task

Participants counted the number of strokes of a kanji character, and answered as quickly as possible after the character appeared on the screen. Kanji stimuli, about 11×11 cm in size and positioned at the center of the screen, remained for 10s, after which time the participants were not allowed to answer. Note that each kanji has many strokes and individuals usually do not remember the number of strokes of a specific kanji but remember the stroke order and the shape of the character. Therefore, they cannot immediately answer the number itself, but they can retrieve it if they have time to call up the image of the character.

#### Procedure

The participants performed both the construction and the stroke count tasks, with the order of the tasks and stimuli sets counterbalanced. During the experiment, the participants sat on a chair with a display on a table placed at about 60cm away from them. They carried out 22 trials for one task, and a total of 44 trials in Experiment 1. The inter-trial interval was about 2s. Before the main trails, they performed two test trials using stimuli which were not included in the main stimuli sets. The participants had breaks for about one minute between the tasks.

The participants were not given any instruction about their hand usage during the trials. No one noticed the purpose of Experiment 1, that is, to investigate whether they showed spontaneous kusho behavior or not. We monitored their hand movement using a video camera to record and confirm the occurrence of kusho behavior.

#### Analysis

We calculated the number of correct responses (CR), and response time (RT) from the end of the last stimulus. Error trials and trials with mechanical errors were excluded in calculating RT (S1 File). Further, the number of occurrences of kusho behavior was counted. Two experimenters watched the participants’ hands and defined whether the participants showed kusho behavior or not. The criterion for the occurrence of kusho behavior was whether or not the participants moved any of their fingers to write any parts of the stimuli presented.

Although the purpose of Experiment 1 was to investigate whether the younger generation today demonstrate kusho behavior or not, *χ* ^2^ test was performed to clarify which task caused more people to use kusho. The number of CR and RT are described but not discussed, as they were not the focus of Experiment 1.

#### Ethics Statement

The present study was conducted in accordance with the principals in the Declaration of Helsinki. The study and consent procedure were approved by the Ethics Committee on Human Research of Waseda University (2012–002). All participants provided their written informed consent.

### Results

**Fig 2:**
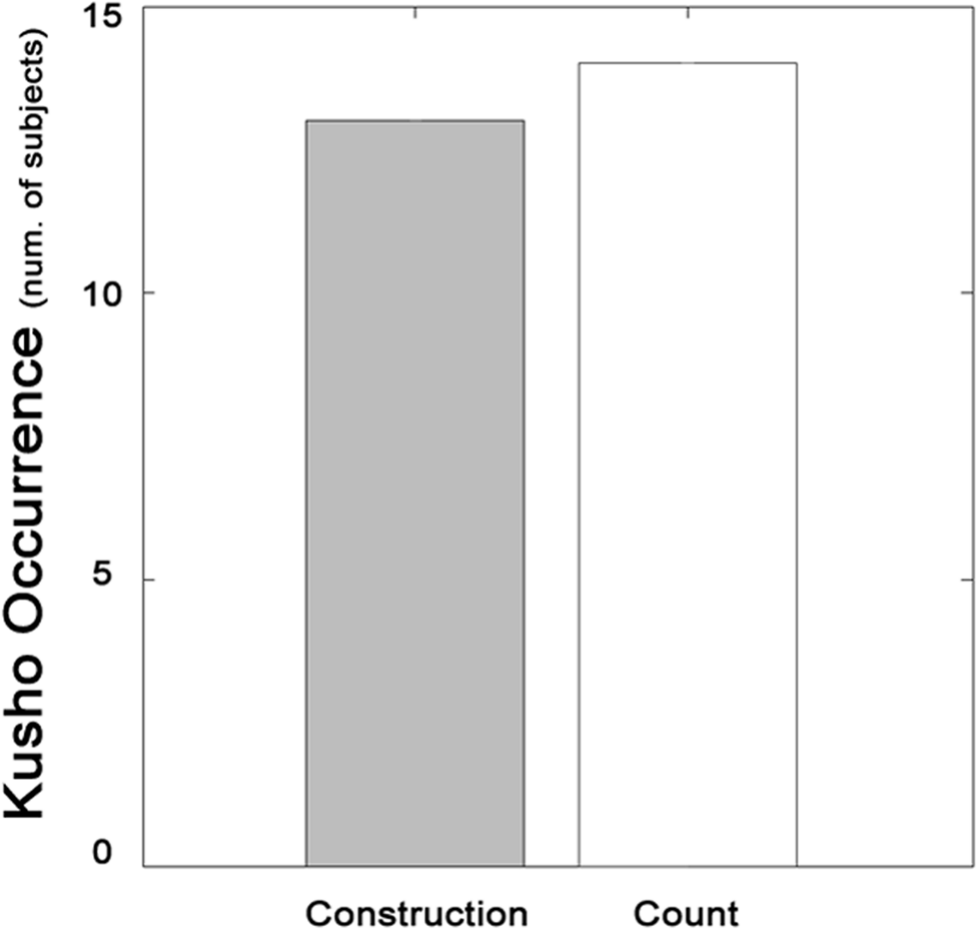
Kusho occurrence in the construction task and the stroke count task. In both tasks, kusho behavior appeared in almost all participants with no difference in the occurrence of kusho behavior between two tasks.

The participants showed kusho behavior with high probability, in the tasks, and the frequency of kusho behavior did not differ between the construction task and the stroke count task (*χ* ^2^(1) = 0, *n*.*s*.). Thirteen out of fifteen participants showed kusho behavior at least once in the construction task, and fourteen out of fifteen participants showed it in the stroke count task (Fig 2). One participant did not show kusho behavior in either task, and the other participant did not show it in the construction task but showed it in all 22 trials in the count task. Seven participants showed it in all trials in both tasks and thirteen participants showed it in all trials in the count task. The mean and SD for CR and RT were 12.7 ± 3.4 and 2710.0 ± 862.3ms for the construction task, and 19.8 ± 1.3 and 5451.6 ± 811.7ms for the stroke counting task.

### Discussion

The results of Experiment 1 confirmed that the participants today demonstrated kusho behavior to solve the construction task, supporting previous studies [1]. Moreover, almost all participants showed kusho behavior in the stroke count task. This finding was in line with Michaux et al. [17], who argued that finger movements could help in procedural cognitive processes. Although counting strokes does not necessarily require finger movement, it would be very helpful to move a finger in the defined stroke order as if actually writing, particularly in order not to miscount or skip a stroke. These findings together suggest that finger movement interacts with the execution of cognitive processes. To discuss what processes were modified by kusho behavior and to define whether kusho behavior worked similarly in both the construction and stroke count tasks, we conducted another series of experiments on the functional role of the behavior.

## Experiment 2

In Experiment 2, we investigated the visual/kinetic effects of kusho behavior on the construction task. In this task, spontaneous kusho behavior occurred when the participants were given no restrictions on hand movements in Experiment 1. Although finger movements themselves could activate kinetic representation of characters and help solve the tasks, the possibility that kinetic aspects of finger movement influence task performance has not been examined so far. In Experiment 2, we introduced hand conditions related to kusho behavior (described below), and a controlled visual condition; participants kept their eyes on the stimuli on a display (Eye-on-display) or their hand (Eye-on-hand). Except for those points, the experimental setups, stimulus sets and methods for Experiment 2 were basically the same as in Experiment 1.

In Experiment 2, we introduced three hand conditions: the Kusho condition, the Still condition, and the Circle condition. In the Kusho condition, participants were allowed to move their right index finger freely in a writing manner as long as the index finger touched the table when they were writing; they could execute kusho behavior. In the Still condition, the participants kept their right fist clenched on the table; they did not move any of their fingers. In the Circle condition, the participants moved their right index finger in a circular manner on the table during the trials; they kept drawing circles. The left hand was always kept fisted on the table in all conditions.

In the following experiments, different sets of participants, who were all Japanese native speakers and had completed their elementary to high school schooling in Japan without any problems in reading and writing Japanese characters, took part in each experiment,

### Methods

Thirty-six right-handed students (20.8 ± 1.9 years old) participated in Experiment 2. They were randomly assigned to one of the two visual conditions: the Eye-on-hand condition or the Eye-on-display condition. They carried out the construction task where the stimuli were presented successively in the three hand conditions (Kusho, Circle, and Still) and were asked to watch either their right hand or the display after the last stimulus disappeared. The order of hand conditions was counter-balanced among participants, and the order of stimulus presentation was randomized. A stimulus set was randomly assigned to each hand condition.

The participants responded to 22 stimuli in each hand condition and 66 stimuli in total. The response time which was limited to 10s started from the end of last stimulus presentation. All the other procedures were the same as in Experiment 1. We conducted a mixed two-way ANOVA (2 visual conditions × 3 hand conditions).

### Results

**Fig 3:**
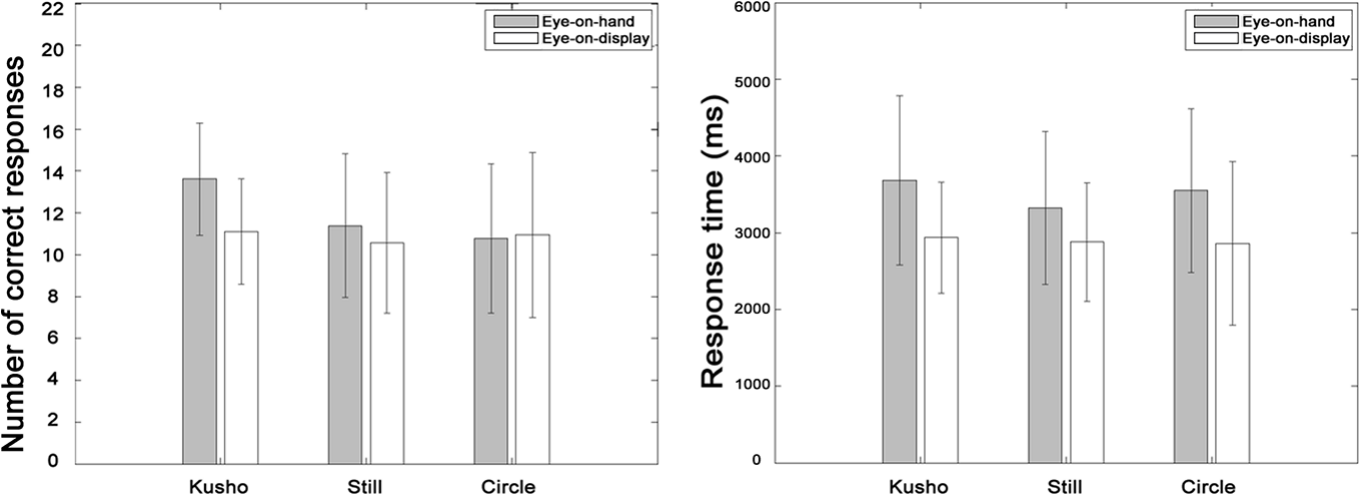
Correct response and response time in Experiment 2. The error bar indicates standard deviation. The number of correct responses in the Kusho condition was significantly larger than in the other two conditions in the Eye-on-hand condition. Response time in the Eye-on-hand condition was significantly longer than in the Eye-on-display condition.

The results showed that number of correct responses in the Kusho condition was greater than in the Still or Circle conditions in the Eye-on-hand condition, whereas there was no effect of hand conditions in the Eye-on-display condition (Fig 3). By contrast, there was no effect of hand condition for response time. In the Eye-on-hand condition, the mean and SD for CR was 13.6±2.8, 11.4±3.5, and 10.8±3.7, and RT was 3682.4 ±1134.9ms, 3324.0±1026.5ms, and 3550.5±1097.3ms. In the Eye-on-display condition, the mean and SD for CR was 11.1±2.6, 10.6±3.5, and 10.9±4.0, and RT was 2937.1±744.7ms, 2877.4±794.1ms, and 2860.6±1099.0ms respectively.

The ANOVA showed a significant main effect of hand condition and an interaction effect between hand and visual conditions for CR (*F*(2,68) = 5.22, *p*<.01.; *F*(2,68) = 3.39, *p*<.05). Simple main effect of the hand condition was found only for the Eye-on-hand condition *(F*(2,34) = 7.88, *p*<.001), and multiple comparisons with Shaffer’s method revealed that CR for the kusho condition was significantly higher than those of the Still condition and the Circle condition(*t*(17) = 3.48, *t*(17) = 3.29, *p*<.05). ANOVA for RT showed that there was a main effect of the visual condition (*F*(1,34) = 6.51, *p*<.05), indicating that RT in the Eye-on-hand condition was significantly longer than in the Eye-on-display condition. No effects related to hand condition were observed.

### Discussion

Experiment 2 supported the idea that visual aspects of kusho behavior facilitate cognitive processing but that its kinetic aspects do not. In the Eye-on-hand condition, the participants made more correct responses in the Kusho condition than in the other two conditions. Executing irrelevant movements did not interfere with the performance. These finding suggest that visual feedback of finger movements in writing facilitated cognitive processes, supporting the visualization hypothesis of kusho behavior.

The delayed response time in the Eye-on-hand condition may stem from the extra movement of participants to turn their face back toward the computer. Response time in the Eye-on-hand condition was longer than that in the Eye-on-display condition. In the Eye-on-hand condition, participants had to reposition their face to answer into the microphone in front of them, which might have caused longer response time in the Eye-on-hand condition in the construction task.

In Experiment 2 kinetic aspects of finger movements did not affect performance in the construction task, although previous studies have suggested the possible contribution of the kinetic aspects of movement (Sasaki 1987). To confirm that the kinetic aspects of finger movements do not affect cognitive processes in the construction task, we carried out Experiment 3 using different versions of the construction task in which participants did not see their hand.

## Experiment 3

The purpose of Experiment 3 was to provide supplementary support for the results in Experiment 2 which showed that the finger movement of kusho behavior itself did not facilitate cognitive performance when participants did not see their finger moving. We carried out two types of construction tasks; one was a replication of Sasaki’s study (Sasaki 1987), and the other was a kanji construction task where kanji parts were presented simultaneously (Matsuo et al. 2001). In the simultaneous presentation task, three types of stimulus presentation time were used to examine any possible effects of kusho behavior on memory retention. Table 1 shows the differences in design of the construction tasks in Experiments 2 and 3.

**Table 1:** Differences in design of the construction tasks in Experiments 2 and 3.

	Experiment 2	Experiment3	
		Replication	Simultaneous
**Hand conditions**	Kusho/Still/Circle	Kusho/Still/Circle	Kusho/Still/Circle
**Visual conditions**	Eye-on-hand/display	Eye-on-display	Eye-on-display
**Presentation time**	10s	30s	1s/3s/10s
**Presentation method**	Successive	Successive	Simultaneous
**Total number of stimuli**	66	12	66

### Methods

#### Replication task in the Eye-on-display condition

Twenty-seven right-handed students (20.4 ± 1.8 years old) participated in the replication task. They were randomly assigned to one of the three hand conditions. Methods used in the experiment followed Sasaki’s study [1] except for the following two points. First, we used a display to provide stimuli while Sasaki’s study used cards. Second, we instructed participants to watch the display during the trials, but Sasaki’s study did not give such instructions to their participants. The stimuli were presented successively with a time limit to answer of 30s. As in Sasaki’s study, we used 12 stimuli in random order. Due to the small number of the stimuli, we used a between-subjects one-way ANOVA (3 hand conditions).

#### Simultaneous presentation task in the Eye-on-display condition

Forty-five right-handed students (21.0 ± 1.3 years old) were assigned to one of three different stimulus presentation time groups (1s, 3s, and 10s). Kanji parts were presented at the same time [23] in a triangular arrangement and disappeared after a certain delay (the stimulus presentation time). The response time was calculated from the onset of stimulus presentation. The time limit to answer was 10s. In the 1s and 3s conditions, random dots appeared as a mask after the stimuli disappeared. All the other methods were the same as in the Eye-on-display condition in Experiment 2. We conducted a mixed two-way ANOVA (3 hand conditions × 3 levels of presentation time).

### Results

#### Replication task in the Eye-on-display condition

**Fig 4:**
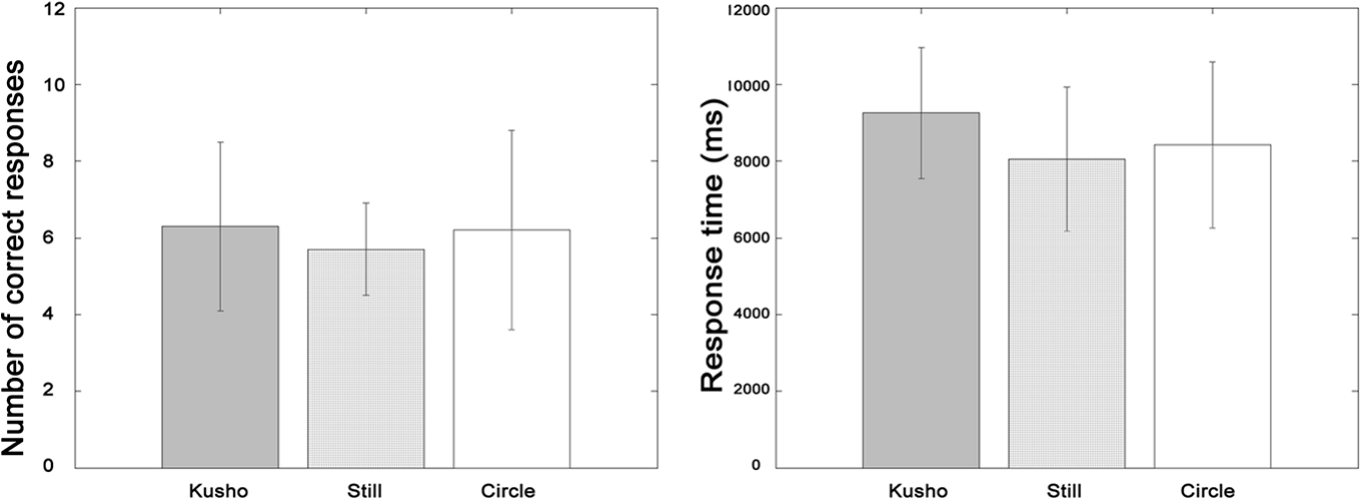
Correct response and response time in the replication task. The error bar indicates standard deviation. There were no significant effects at all.

The results showed that there was no effect of hand condition on performance of the construction task in the Eye-on-display condition (Fig 4), despite using the same methods and stimuli as Sasaki’s study. The mean and SD for CR was 6.3±2.2, 5.7±1.2, and 6.2±2.6, and RT was 9253.7±1709.9ms, 8053.5±1886.2ms and 8420.0±2170.0ms. Significant main effect of hand condition was not observed for either CR (*F*(2.24) = 1.148, *n*.*s*.) or RT (*F*(2.24) = 0.811, *n*.*s*.).

#### Simultaneous presentation task in the Eye-on-display condition

**Fig 5:**
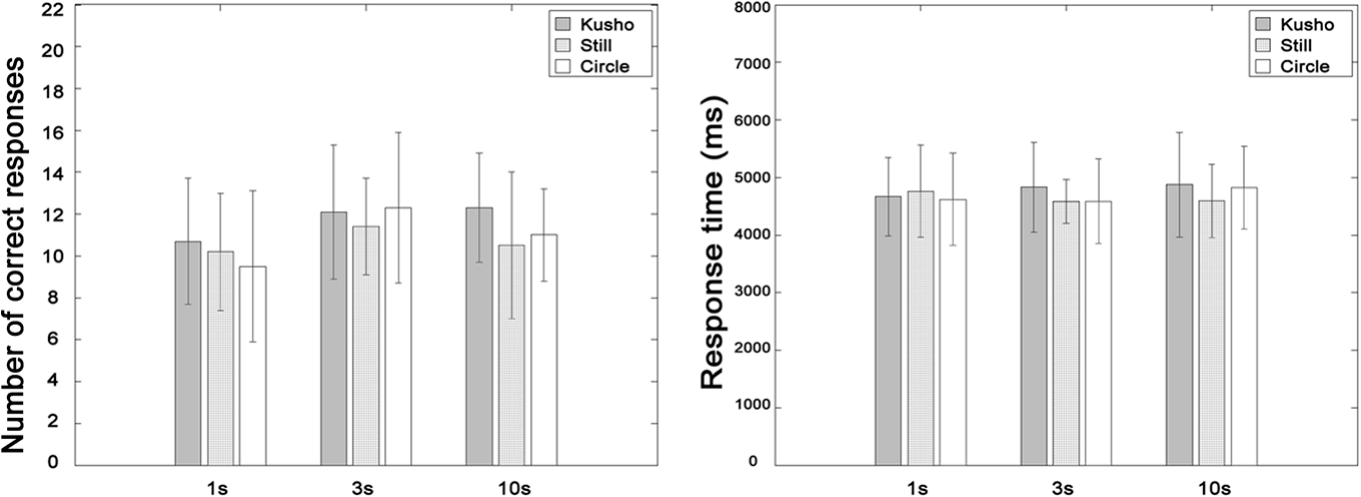
Correct response and response time in the simultaneous presentation task. The error bar indicates standard deviation. There were no significant effects at all.

The results showed that there were no effects of either hand condition or presentation time on performance of the construction task using simultaneous stimulus presentation in the Eye-on-display condition (Fig 5). The CR averages were 11.7, 10.7, and 10.9, and RT (Pooled with presentation time) was 4790.0ms, 4645.3ms, and 4673.4ms for the Kusho, Still, and Circle conditions. Details are shown in Table 2. No statistically significant effects of hand condition and presentation time were observed for either CR (*F*(2.42) = 1.934, *n*.*s*.; *F*(2.84) = 2.549, *n*.*s*.; *F*(4.84) = 0.935, *n*.*s*.) or RT (*F*(2.42) = 0.148, *n*.*s*.; *F*(2.84) = 0.536, *n*.*s*.; *F*(4.84) = 0.520, *n*.*s*.).

**Table 2:** Number of correct response and Response time (ms) in the simultaneous presentation task.

**CR**	**Kusho**	**Still**	**Circle**
**1s**	10.7 ± 3.0	10.2 ± 2.8	9.5 ± 3.6
**3s**	12.1 ± 3.2	11.4 ± 2.3	12.3 ± 3.6
**10s**	12.3 ± 2.6	10.5 ± 3.5	11.0 ± 2.2
**RT**	**Kusho**	**Still**	**Circle**
**1s**	4665.0 ± 676.9	4761.9 ± 803.4	4616.5 ± 801.5
**3s**	4831.3 ± 779.5	4584.1 ± 380.7	4583.3 ± 735.7
**10s**	4873.7 ± 910.8	4591.3 ± 639.8	4820.4 ± 716.5

### Discussion

Experiments in the Eye-on-display condition provided three main findings. First, there was no significant effect of finger movements on performance in the cognitive task, despite using the same stimuli as Sasaki’s study [1]. This result suggests that the kinetic aspects of finger movements neither improve nor degrade the accuracy and speed of cognitive processing. The Eye-on-display condition was assumed to involve not visual feedback but kinetic feedback from kusho movements. Second, the differences in presentation time did not yield any statistically significant effects on performance of the construction task, indicating that the kinetic aspects of finger movements might not be useful in maintaining memory traces of stimuli. Although the null results themselves do not suggest that there were no effects of kinetic aspects of finger movements on cognitive processing for solving the construction tasks, it would be beneficial to discuss them at least in comparisons with other similar experiments. Accordingly, we advance the discussion based on the null results with limited implications.

The different versions of the construction task in Experiment 3 confirmed the results of Experiment 2; we did not obtain any facilitation effects of kusho behavior in the Eye-on-display condition. These results were not accordance with Sasaki’s report [1]. Although the Kusho conditions showed consistently larger numbers of correct responses than the Still conditions in the present experiments, they were not statistically significant nor did they reach the level of correct responses in the kusho condition in the Sasaki’s study. The previous kusho condition yielded almost twice as many correct responses as those of the corresponding still condition. This discrepancy might be partially attributed to visual conditions; participants in Sasaki’s study [1] possibly saw their hand, whereas participants in the current experiments in the Eye-on-display condition did not see their hand. Although the cause of the discrepancy in results between the studies could not be clarified, the results of Experiments 2 and 3 support the idea that the visual feedback of hand movement is important in solving construction tasks.

## Experiment 4

In Experiment 4, we investigated the visual/kinetic effects of kusho behavior on a stroke count task, which also elicited spontaneous kusho behavior in Experiment 1. Like Experiment 2, we introduced three hand conditions and set two visual conditions. Except for these points, the experimental setups, stimulus sets and methods were essentially the same as those for Experiment 1.

### Methods

Thirty-six right-handed students (22.0 ± 2.1 years old) participated in the stroke count task. The participants carried out the stroke count task in the three hand conditions (Kusho, Circle and Still) and in two visual conditions (Eye-on-hand and Eye-on-display). The order of the hand condition was counter-balanced among participants. We conducted a mixed two-way ANOVA (2 visual conditions × 3 hand conditions).

### Results

**Fig 6:**
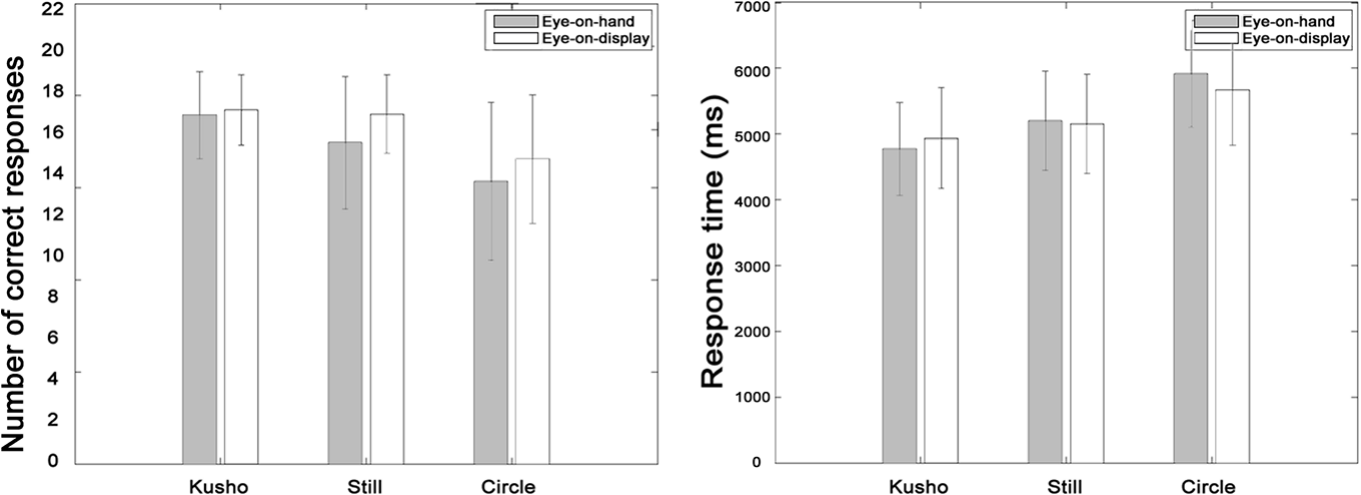
Correct response and response time in the stroke count task. The error bar indicates standard deviation. CR and RT of all hand conditions significantly differed from each other. There was no significant difference between the visual conditions.

The results showed that the number of correct responses and response time were the greatest and the shortest in the Kusho condition, followed by the Still condition and the Circle condition in the two visual conditions (Fig 6). In the Eye-on-hand condition, the mean and SD for CR was 19.0±2.4, 17.4±3.6, and 15.3±4.3, and RT was 4773.5 ±706.5ms, 5203.3±753.6ms, and 5919.1±809.3ms. In the Eye-on-display condition, the mean and SD for CR was 19.2±1.9, 19.0±2.1, and 16.6±3.5, and the RT was 4939.5±771.8ms, 5152.8±750.9ms, and 5666.5±838.8ms respectively.

The ANOVA showed a significant main effect of hand condition in CR (*F*(2,68) = 16.68, *p*<.001) and RT (*F*(2,68) = 35.23, *p*<.001). The interaction effect between the hand and visual conditions was not significant for either CR or RT. Multiple comparisons with Shaffer’s method revealed that there were significant differences between conditions for CR (Kusho>Circle: *t*(34) = 4.95, Kusho>Still: *t*(34) = 3.74, Still>Circle: *t*(34) = 2.06, *p*<.05) and RT (Kusho<Circle: *t*(34) = 6.96, Kusho<Still: *t*(34) = 5.53, Still<Circle: *t*(34) = 3.58, *p*<.05).

### Discussion

The results showed both facilitation and interference effects of finger movement regardless of the visual condition in the stroke count task, suggesting that kinetic information rather than visual information contributes to the stroke counting processing. Moving a finger as if one wrote characters improved both CR and RT, whereas unrelated finger movement disturbed the performance compared to the static hand condition. This effect implies that kinetic feedback from finger movement is used as a counter in stroke counting in the central system. By contrast, the visual condition did not affect performance in the task, suggesting that visual information is not involved in a counting task.

Unlike the construction task, the stroke count task did not show any difference in response time between the visual conditions. This discrepancy between the two tasks might be due to the predictability of the timing of answer in each task. In the construction task, participants do not know when they will come up with the answer, whereas in the counting task, they can predict when the finger writing ends. In the latter condition participants could turn their face back to the microphone before the finger movement finishes. This advantage might have allowed them to answer more quickly in the Eye-on-hand condition eliminating the difference between the visual conditions in the stroke count task. However, this possibility cannot be proved as we did not record any motion data about face return timing.

## General Discussion

The present study aimed to confirm the existence of kusho behavior in the individuals today and to reveal its functional role on cognitive processing. Experiment 1 revealed that young Japanese people today still demonstrate kusho behavior when they try to solve cognitive tasks related to kanji character. Experiment 2 showed that moving a finger as if one wrote characters improved the correct response rate in the kanji construction task only when the participants observed their finger moving. This result indicates that information from the kinetic aspects of kusho behavior was not enough to improve cognitive performance. Experiment 3 replicated the lack of facilitation effect associated with the kinetic aspects of kusho behavior, showing that none of the hand conditions had an effect on task performance when participants did not see their hands in the construction tasks. Experiment 4 observed both facilitation and interference effects of finger movement in the stroke count task. These results suggest that kusho behavior has different functional roles in the two types of cognitive tasks; visual aspects of kusho behavior contribute to the construction task, while the kinetic aspects of kusho behavior help to execute the stroke count task.

### Recalling characters based on visual information

The present results indicate the functional role of kusho behavior on character recall more clearly than previous studies, supporting the visualization hypothesis for the role of kusho behavior in a construction task. Sasaki [1] implied that individuals employ kusho behavior to visualize characters and suggested that the kinetic aspects of kusho could enhance the recall of characters. To test these hypotheses, we introduced a kinetically unrelated movement (the Circle condition) and a situation in which participants did not see their finger movements (the Eye-on-display condition). Our results showed that the trigger of the kusho effect was to see finger movements during the solution of the construction task. This finding supports the visualization hypothesis rather than the kinetic facilitation hypothesis, providing evidence of interactions between cognitive processes and body actions.

The visualization effect of finger writing is consistent with previous studies on healthy individuals as well as neuropsychological patients. Yim-Ng et al. [21] reported that kinetic information without spatial information impaired character recognition. In their experiment, blindfolded participants traced a kanji character guided by an experimenter, and were asked to identify the character. When they traced a character with an altered spatial configuration but with a not-altered stroke sequence, they performed wores than with a preserved spatial configuration. Kinetic information from writing movements is as well preserved if the stroke sequence is not changed. This result indicates the importance of a visual image of a character for recalling it. It is also known that some alexic patients could read a character by observing the writing behavior of another person [27]. Tanaka et al. [28] reported on a patient with a lesion of the splenium of the corpus callosum, whose reading was severely impaired in normal conditions but drastically improved when he observed the writing actions of others. Although the exact mechanism of this neuropsychological phenomenon is still unclear, there might be neural substrates subserving character recognition processes which are driven by visual observation of writing movements.

The visualization effects of kusho behavior can be interpreted within a framework in which visual feedback is superior to kinetic feedback in action/character recognition. In terms of computational motor control, visual feedback might be a more appropriate signal to make a prediction in a forward model rather than kinetic feedback or motor commands for kusho behavior. This possibility agrees with the idea of shared neural substrates between action execution and observation, which are sometimes assumed to be mirror neurons [29–32]. Based on this idea, there would be a system matching action observation and execution [33]. Moreover, imitation based on observation is easier than action based on instructive cues in finger movements [34]. Accordingly, in action/character recognition, visual feedback might be effective information for the activation of the representation of a specific character.

Nevertheless, the current finding does not exclude the possibility of kinetic effects of kusho behavior, although there was no facilitation effect on the cognitive tasks. Healthy individuals and even alexic patients can recognize a character which is kinetically presented via passive finger movements [10,35]. These studies indicate that we can recognize characters based on kinetic information, as well as that there might be several distinct routes to identify characters using either visual or kinetic information. It cannot be denied that the kinetic aspects of kusho behavior might contribute to a recall process such as the construction tasks in the present study.

There still remains the question of why kusho behavior occurs so often despite its very limited effect on solving tasks. Kanji-culture individuals demonstrate finger writing on almost every surface: on tables, the palm of their other hand, their knee, and even in the air [1]. The present experiments revealed that kusho behavior had a facilitation role only when the participants could see their finger movements, and the amount of facilitation was relatively small. Although there is a possibility that long-term practice of character writing in childhood elicits spontaneous writing actions, it seems strange that such an ineffective strategy is used so frequently and universally.

One possible reason for the frequent use of kusho behavior is provided in terms of the relationship between task performance and brain activation. Kinetic effects of kusho behavior were not observed when the participants did not see themselves use kusho. However it is possible that kinetic information from kusho behavior reduces neural loads on cognitive processing. That is, the neural load on brain is distributed and less intense in the area responsible for the particular brain function being used at the time. This situation may increase neural health but does not necessarily change cognitive performance itself. Matsuo et al. [23] has reported that the brain areas for kusho are the boundary area between the inferior parietal lobule and occipital lobe. Taken together with this report and our speculation, the activation level of the parieto-occipital area would decrease when the participants employ kusho behavior during a construction task while task performance would remain the same. In addition, our findings also predict that task performance would be positively related to activation of the visual area evoked by observation of kusho behavior. Future studies are needed to reveal a clear relationship between task performance and brain activities modulated by finger movements.

### Counting numbers based on kinematic information

The present study also reported kusho effects on counting the number of strokes of a character, which is often observed in a daily life but has rarely been investigated in previous studies. The results showed the facilitation of the Kusho condition as well as interference effects of the Circle condition regardless of visual condition, supporting the kinetic facilitation hypothesis for a stroke count task.

The results indicated that the kinetic information from finger movements for counting the strokes of a character help in its sequential processing. In both visual conditions, employing kusho resulted in more correct responses and a faster response time, while unrelated movements degraded the performance. It is likely that counting strokes requires sequential mental confirmation of the number of strokes and that finger writing ensures that the counting as writing movement is essentially a one-way trajectory. Unrelated movements might confound the sequential processes whereas a motionless hand might not disturb them. Matsuo et al. [22] reported that activation in the dorsal occipitoparietal areas and the primary visual area decreased when participants used kusho in a counting task. That is, instead of the visual aspects, the kinetic and sequential aspects of kusho help carry out the process of counting. This is in accordance with our results; both suggest that central processing depends on kinetic information rather than visual information in a counting task when participants are executing finger movements.

The positive and negative influence of finger movement on stroke count task performance may also support the kinetic facilitation hypothesis. Finger writing can confirm the progress of the counting in a gradual manner based on the discrete information of each stroke of a character. By contrast, circular movements provide unrelated kinetic signals which could not match the progress of counting, and seemed to disturb the continuous kinetic feedback required to make one-to-one correspondences in a sequential task. Such kinetic interactions of finger movements agree with the idea of a motor contribution to semantic processing [13,36] or ‘embodied cognition’, which emphasizes the role of sensory and motor functions in cognition itself [37,38]. Although the unclear influence of kusho behavior on cognitive processing makes caution in drawing hasty conclusions, the present results at least suggest that the facilitation and interference effects in counting strokes of a character can be attributed to the kinetic aspects of kusho behavior.

We should also separately consider the origin of kusho behavior from its functional roles. The occurrence of the behavior observed in the present study may be attributed to repeated writing when learning characters. Sasaki [1] suggested that writing experience could cause the coupling of a motor representation with a character representation. Siok et al.’s [39] imaging study of Chinese speakers suggested the importance of cultural factors for the involvement of writing abilities in reading processes. Such cultural learning styles may contribute to the spontaneous occurrence of finger movements, and possibly cause kinetic facilitation effects of finger movements on recalling a character. However, such visible behavior may not necessarily have a functional role in the central processing required to solve the task. The present results showed that the kinetic aspects of finger movements were likely related to their occurrence but did not influence the recall process. Accordingly, we should be careful to distinguish between the origin of finger movement and its functional role.

Kusho behavior for counting should also be distinguished from hand gestures for numbers, although they are similar to each other in that they could interact with numerical processing, which was also observed in the present study. Hand gestures are associated with a particular number in a one-to-one manner, while movements for counting the number of strokes do not. Rather, finger movements for reconstructing a kanji character might be captured in terms of the item-specific memory. That is, kusho behavior to retrieve internal representations may involve one-to-one memory processing, which is often assumed in fields which study the relationship between mathematical ability and body action [40]. It is also in agreement with the idea that only body gestures which are specifically associated with memory representations can lighten cognitive loads [41–42]. The current study demonstrated that very similar finger movements differently influence recalling and counting processes, depending on the available modality. The results are important to further clarify the nature of habitual gestures.

## Conclusion

The present study demonstrated that kusho behavior has different functional roles in the two types of daily tasks, recalling characters and mentally counting strokes. First, the results revealed that the visual aspects of finger writing improved task performance in character recognition. The visual information from a moving finger likely facilitates identifying a character, whereas the kinetic behavior itself may involve neither solving the task nor retaining a memory trace. The role of finger movement in visualizing kanji might shed new light on neuropsychological phenomena in brain-damaged patients such as kinesthetic facilitation of trace reading. Second, the present study found that in the stroke count task kinetic aspects of finger movements helped or hindered the counting processes depending on the types of movement: kusho behavior improved performance and unrelated movements degraded it, regardless of visual conditions. These modality dependent characteristics of kusho effects suggest the mechanism of the particular writing behavior in cognitive processes, which might lead to better understanding of the complex interaction between cognition and action.

## Supporting Information

S1 File Raw RT data. (XLS)
